# People with diabetes foot complications do not recall their foot education: a cohort study

**DOI:** 10.1186/s13047-018-0255-4

**Published:** 2018-04-06

**Authors:** Julia Yuncken, Cylie M. Williams, Rene Stolwyk, Terry P. Haines

**Affiliations:** 1 Peninsula Health, Community Health, PO Box 52, Frankston, VIC 3199 Australia; 2 Department of Physiotherapy, Monash University, McMahon’s Rd, Frankston, VIC 3199 Australia; 3 School of Psychological Sciences, Monash University, Frankston, Australia; 4 Monash Institute of Cognitive and Clinical Neurosciences, Frankston, Australia; 5 Monash-Epworth Rehabilitation Research Centre, Frankston, Australia; 6 Monash Health, Allied Health Research Unit, Kingston Centre, Warrigal Rd, Cheltenham, VIC 3192 Australia; 7 Monash University Department of School of Primary and Allied Health Care, McMahon’s Rd, Frankston, VIC 3199 Australia

**Keywords:** Podiatry, Diabetes education, Foot education, Education retention

## Abstract

**Background:**

The purpose of this study is to document what and how diabetes specific foot health information was provided during a podiatry consultation, and what information was retained at 1 month post consultation.

**Methods:**

This project was embedded within a prospective cohort study with two groups, podiatrists and people with diabetes. Data collection included the Problem Areas in Diabetes Questionnaire (PAID), Montreal Cognitive Assessment (MoCA), information covered during the consultation, method of delivery and perceived key educational message from both participant perspectives at the time of the appointment and 1 month post appointment.

**Results:**

There were three podiatrists and 24 people with diabetes who provided information at the two time points. Diabetes education provided by the podiatrists was mostly verbal. The key educational message recalled by both groups differed at the time of the appointment (14 out of 24 of responses) and at 1 month post the appointment time (11 out of 24 of responses).

**Conclusions:**

Education is a vital component to the treatment regime of people with diabetes. It appears current approaches are ineffective in enhancing understanding of diabetes impact on foot health. This study highlights the need for research investigating better ways to deliver key pieces of information to this population.

**Electronic supplementary material:**

The online version of this article (10.1186/s13047-018-0255-4) contains supplementary material, which is available to authorized users.

## Background

It is estimated that 415 million people in the world are currently living with diabetes [1]. Those with diabetes have a risk of lower limb amputation 25 times greater than those without diabetes [1]. Diabetes Mellitus is a chronic disease that has ongoing health, social and economic burdens across society [1]. In Australia, it estimated to affect 7% of the adult population with 15% of those to develop a foot complication over their lifetime [1]. The annual cost of diabetes in Australia is estimated at $4025 per person with no diabetes complications, this increases to an estimated annual cost of $9645 for someone with diabetes and complications [2].

Foot complications arising from diabetes can range from neuropathy, vascular disease, ulceration, infection and amputation [3]. The International Diabetes Federation diabetes education advocates for diabetes education as an integral part of the overall treatment plan to prevent long term disease complications [1]. Podiatrists play a large role in education for the prevention of foot complications in people with diabetes. Subsequently, podiatrists also regularly teach self-management strategies for people with or without foot complications relating to their diabetes [4]. Current diabetes guidelines recommend education provision, however, there are no clear guidelines on what information should be covered in a consultation and what is the most effective education medium (i.e.: Written, verbal, technology aided) [3].

This study aimed to document how and what diabetes specific foot health information was provided during podiatry consultations and what information was retained 1 month post consultation.

## Methods

### Design

This was a cohort study. People with diabetes were provided with diabetes education, within a podiatry appointment, and followed up 1 month later. The Human Research Ethics Committee of Peninsula Health approved this study (LRR/14/PH/14).

### Participants and setting

This study was located within the Peninsula Health podiatry department, Victoria, Australia. The podiatry department has four community health sites and four hospitals. People who attend the podiatry service are triaged according to the Department of Health and Human Services – Community Health Priority Tool [5]. This tool indicated the priority of people based on their health status and presenting complaint. People are considered a medium priority, if they have never had a diabetes foot health assessment or if they had, did their foot/leg problem interfere with their ability to work, act as a carer, participate in normal daily activities [5].

There were two paired groups of participants recruited for this study. Podiatrists were eligible to participate if they worked within the Podiatry Department and currently assessed people with diabetes. People over the age of 18, attending the podiatry department and having a diagnosis of Diabetes Mellitus (type 1 or 2) were invited to participate if they had a consultation with a participating podiatrist. People were ineligible to participate if they were unable to read English or exhibited significant cognitive impairment on a cognitive screening tool (Montreal Cognitive Assessment score (MOCA) ≤26).

### Outcome measures

The primary outcome measure was patient participant-podiatrist concordance in response to the question “What was the key message from the consultation?” This question was verbally asked by researcher JY, of both participant pairs following the consultation, and of the patient participant 1 month following the consultation. The study’s principal researcher (Rater 1) and a podiatrist who was not one of the study investigators (Rater 2) separately examined both the patient participant response and corresponding podiatrist response and classified this dyad as being in “full agreement”, “partial agreement”, or “no agreement”.

Patient participants were phoned at 1 month following the consultation to collect the secondary outcome measure. These included qualitative responses about general diabetes knowledge such as “Can you tell me in your own words how diabetes can affect your feet?”, “Thinking about your consultation and your foot health, what did the podiatrist tell you about diabetes and problems with blood flow?” and “Thinking about your consultation and your foot health, what did the podiatrist tell you about diabetes problems with feeling in the feet?”

### Demographic measures

Demographic data was collected from the participants including age, gender, year left school, recency of diabetes diagnosis, Montreal Cognitive Assessment (MOCA) score, Problem Areas in Diabetes (PAID) scale and modality of podiatry provided education within appointment.

The MOCA was used as a measure of baseline cognitive function for the participants attending the podiatry appointment to gain an understanding of any cognitive decline. Participants were included if they had a score greater than 26 as under this score was indicative of cognitive decline [6]. The MOCA also allows for adults who were early school leavers within the scoring system.

The PAID scale measures a persons’ diabetes related emotional distress. It correlates with concepts such as social support, coping style, depression, future blood glucose control and health beliefs [7]. The PAID scale contains 20 questions relating to negative emotions in regards to the personal impact of diabetes. Once completed, a score is calculated between 0 and 100, a score of 40 or higher may indicate emotional burnout whilst an extremely low score (≥10) in conjunction with poor blood glucose control may indicate denial regarding their diabetes [7].

The modality of education delivery employed by the podiatrist was categorised by the researcher during the observed podiatry consultation. The categories included 1) Written only, 2) Verbal only and 3) Written and verbal.

Demographic data collected from the podiatrists included age, recency of practice, level of university education and seniority in health care service.

### Procedure

Podiatrists were recruited through an email to the entire department and provided informed consent to participate. At the time of the study, there were 13 podiatrists of varying experience within the department. Podiatrists were made aware of the study purpose and instructed to maintain their usual practice of diabetes education during consultations. Eligible patient participants were approached in a sequential order according to their presentation and screened against the study inclusion criteria. Following inclusion, the PAID questionnaire was completed and the podiatry appointment commenced. There was no effort to standardise the podiatry appointment. During the appointment, the researcher silently observed and recorded what information relating to diabetes was provided and how it was delivered, oral or written or both. This was recorded via a tick the box data collection sheet. Data collection sheet (Patient Participant Checklist) included content and how it was delivered. This data collection sheet was standardised to each observation (Additional file 1). At the conclusion of the appointment, podiatrists and the participating patient were separately asked the question “What was the key message from the consultation?” This information was also collated on the ‘Patient Participant Checklist’ (Additional file 1).

One month following the appointment, each participant was contacted via the telephone. Participants were again asked the same question “What was the key message from the consultation?”

### Data analysis

Paired participant responses to the question about the key message (both at baseline following the appointment and 1 month later) were written verbatim by the principle researcher while in person with the podiatrist and patient participant or on the phone with the patient participant. Comparisons were made between the patient participant’s baseline response and the podiatrists baseline response, the patient 1 month recall response and the podiatrist baseline response, and the patient baseline and 1 month recall responses. A weighted kappa [8] was calculated with Stata 13 to determine the level of agreement between the paired responses. For each rater, the proportion of responses falling into each category was calculated along with the binomial 95% confidence intervals. A post-hoc power analysis indicated that a sample size of 24 participants would provide a 95% confidence interval of +/− 0.21 for the proportion of subjects within a particular agreement category (eg. full agreement, partial agreement, no agreement) if the proportion within a particular category was 0.50. To minimise podiatrist bias, three podiatrists were recruited and each paired with eight patient participants to achieve the target sample size.

Thematic analysis was used to derive patterns within the responses from the patient group. Responses collected verbatim at the 1 month post appointment telephone interview were grouped into categories by the lead researcher. All responses from the questions “Thinking about your consultation and your foot health, what did the podiatrist tell you about diabetes and problems with blood flow?” and “Thinking about your consultation and your foot health, what did the podiatrist tell you about diabetes problems with feeling in the feet?” were categorised. Like categories were then grouped into major themes [8].

## Results

There were three podiatrists recruited to the study. They had between 1 and 11 years’ experience, with a mean (SD) age of 31 (4.58) years. One had only worked in community health during their career, one had experience across community health, hospital and private practice settings and the other had worked in community health and private practice. There were 59 potential patient participants sequentially approached, 18 scored below the required MOCA score for inclusion, 14 declined and three cancelled their appointment with the podiatrists on the day they were scheduled for participation. This resulted in 24 patient participants included in the study.

Table 1 displays the patient participant demographic data. The most common diabetes treatment modality was a combination of oral hypoglycaemic medications and Insulin (*n* = 11). The mean (SD) age of the participants was 61 (10.8) years, whilst the average year of school leaving was year 10.3 (1.3) with a range of school completion from year 7 through to completion of undergraduate university degree.

**Table 1:** Patient Demographics and PAID scores

Patient Demographics	Mean (SD) or n(%)
Age	61.0 (10.8)
Gender (male)	15 (63%)
Type of Diabetes (Type 2)	23 (96%)
Duration (years)	15.1 (12.1)
Treatment type	
Insulin	5 (21%)
Oral hypoglycaemic medication	5 (21%)
Oral hypoglycaemic medication + insulin	11 (46%)
Diet only	3 (12%)
Year of completed schooling	10.3 (1.3)
Number of Podiatry consults in the past 12 months:	
*1–2 consultations*	4
*3–5 Consultations*	7
*6–10 Consultations*	3
*10+ Consultations*	10
PAID Score ranges	
*Denial (0–10)*	10(42%)
*Accepting (11–39)*	12 (50%)
*Burnout (40–60)*	2 (8%)

Diabetes related foot health education was provided verbally in all cases. Two patient participants also received hand written personalised information at the end of their consultation. Each consultation room had a brochure stand with current, plain English diabetes and foot health related tri-fold handouts, however none of these handouts were used for any patient participants. The verbatim paired key educational messages were compared from the initial appointment and at one month post appointment (Table 2). The collated and themed responses were rated as ‘full agreement’, ‘partial agreement’ and ‘no agreement’ between the podiatrist and patient participant (Table 3). A Weighted Kappa was used to determine the level of agreement with the ranges The weighted Kappa indicates slight agreement ranging between 0 and 0.20, fair agreement ranging between 0.21–0.40, moderate agreement ranging between 0.41–0.60, substantial agreement ranging between 0.61–0.80 and almost perfect agreement ranging between 0.81–1 [8]. The agreement (weighted kappa) between Rater 1 and Rater 2 was Kw = 0.73 for the baseline patient-podiatrist dyad responses (substantial agreement), 0.78 for the 1 month patient recall-podiatrist baseline dyad (substantial agreement), and 0.60 for the patient baseline-patient 1 month recall dyad (moderate agreement). The themes most commonly highlighted as a key message by the podiatrist was foot inspections and who to contact if the patient participant had any foot related concerns or issues. The patients identified regular foot inspections and footwear as the primary categories of education.

**Table 2:** Content of key educational messages

Key message theme from podiatrist	Number of times coded by *Rater 1*	Number of times message was not reported by patient at baseline, when it was reported by podiatrist according to *Rater* 1	Number of times this message was not reported by patient at 1 month, when it was reported by podiatrist according to *Rater* 1	Number of times coded by *Rater 2*	Number of times this message was not reported by patient at baseline, when it was reported by podiatrist according to *Rater* 2	Number of times this message was not reported by patient at 1 month, when it was reported by podiatrist according to *Rater* 2
Monitor blood sugar levels	11	9	8	11	9	9
Wear appropriate footwear	4	0	2	4	3	3
Foot inspections	15	11	7	14	7	6
Wound care	10	6	8	10	5	6
Who to contact if concerns/issues	12	11	12	11	3	4
Referrals	2	0	2	3	8	8
Orthotics	1	0	0	1	1	0
Other	4	2	1	4	7	6

**Table 3:** Frequency of classifications of each rater of 1. patient and podiatrist responses at baseline, 2. Patient recall responses at one month and podiatrist responses at baseline, and 3. Patient responses at baseline and patient recall responses at one month

	Podiatrist versus Patient (baseline) *Rater 1* N, Proportion (95%CI)	Podiatrist versus Patient baseline *Rater 2* N, Proportion (95%CI)	Podiatrist baseline versus patient 1 month Rater 1 N, Proportion (95%CI)	Podiatrist baseline versus patient 1 month Rater 2 N, Proportion (95%CI)	Patient versus patient 1 month Rater 1 N, Proportion (95%CI)	Patient versus patient 1 month Rater 2 N, Proportion (95%CI)
Full agreement	6, 0.25(0.10, 0.47)	3, 0.13 (0.03, 0.32)	6, 0.25,(0.10, 0.47)	8, 0.33, (0.16, 0.55)	10, 0.42 (0.22, 0.63)	12, 0.50 (0.29, 0.71)
Partial agreement	4 0.17,(0.05, 0.37)	8, 0.33, (0.16, 0.55)	7, 0.29, (0.13, 0.51)	3, 0.13, (0.03, 0.32)	2, 0.08, (0.01, 0.27)	2, 0.08 (0.01, 0.27)
No agreement	14, 0.58 (0.37, 0.78)	13, 0.54, (0.33, 0.74)	11, 0.46, (0.26, 0.67)	13, 0.54, (0.33, 0.74)	12, 0.50, 0.29, 0.71)	10, 0.42,(0.22, 0.63)

Comparison of podiatrist and patient participant responses were poor in both the initial response at the time of the appointment and at the 1 month follow up with only 25% responses in full agreement at both time points. The highest level of agreement was found between the patient at the time of the appointment and the patient response at the 1 month follow up, 41%. No agreement was found between the podiatrist and the patient 58% of the time, at the time of the appointment and 45% of the time at the 1 month follow up. These values are reported within Table 3.

The secondary outcomes highlighted several themes in the responses elicited from patient participants when asked “Thinking about your consultation and your foot health, what did the podiatrist tell you about diabetes and problems with blood flow?” and “Thinking about your consultation and your foot health, what did the podiatrist tell you about diabetes problems with feeling in the feet?” Four major themes were identified as being difficulty understanding physiological concepts, repetition causing inattention, lack of recollection and threat appraisal prompting action.

Many patient participants were aware of the risks and understood how it related to their health, these were categorized as threat appraisal prompting action. The perceived, or unperceived, threat appraisal of diabetes complications of the individual patient may be a contributing factor in the risk of complications or adherence of lifestyle changes for those with diabetes [9]. Ten participants had responses that were categorized into this theme. This included responses like *“It can cut the circulation off which can cause you to lose a limb/Pins and needles, things like that”*. The patient also commented that they would *“Take care of it (feet), clean it”* indicating that they would take action on the education provided. These responses suggested that the patients had an awareness of the problems that they faced and were able to draw a connection to actions that might mitigate this risk. This indicates that the education provided may have increased patient knowledge and awareness of potential problems, of potential solutions, and/or the links between these, though it was unclear whether this information and connection was new to the patient based upon the most recent consultation with the podiatrist.

Several patient participants noted that they either did not receive education or that they could not remember the education provided by the podiatrist during their appointment just one month prior. Four participants responded with noting they had received no education at all *“On the last visit nothing”*. Whilst seven patients responded that they could not remember any education being provided *“Well no, not that I can remember. I know that you have to have good blood flow to the feet”*. These responses indicate that participants either did not absorb any information relating to podiatrist driven education during the consultation, a lack of education provided by the podiatrist, or that the participant did not realise that they were receiving education messages and only absorbed information at a subconscious level.

Of the 24 recruited patients, two participants did not understand the educational message. These participants were aware of the educational message provided by the podiatrist, but did not understand how it related to their personal situation. “*It has been explained but the actual link between metabolism and sugar and circulation to the extremities. I don’t fully understand”*. These responses indicate that some information may have been absorbed during the consultation, but with insufficient clarity for the participant to understand the full picture.

The final theme was about repetition resulting in ambivalence. One participant had a disengaged attitude towards education that he was receiving, *“I got the spiel but you sort of turn off as I’ve heard it a 1000 times. The blood sugars speech and all that”*. It is unclear if this participant understood the education that was provided or not, but indicates that there is potential danger in providing an educational message that the participant has previously been exposed to without checking whether they had already heard and understood this message.

## Discussion

Many of the patient participants recruited to this study did not agree or only partially agreed with the podiatrists key educational message. Many factors may have contributed to this, such as, being an early school leaver, different health priorities, educational methods, or volume of provided information. The three podiatrists that volunteered for the study had varied experience and this was representative of podiatrists working within the public sector in Victoria, Australia.

Of interest within this study, was that many potential participants were ineligible based on the required minimum MOCA score (31%). This was not an unusual finding in studies of people with diabetes [10, 11], one large study finding cognitive impairment and depressive symptoms in older people with diabetes in a cohort of 529 subjects over 70 years of age with diabetes [10]. Within this study, only 36% were free from any cognitive or depressive. Cheng et al. [11] found in their meta-analysis that those with diabetes were more likely to suffer from cognitive impairment than those without. These commonly found cognitive deficits highlight the need for ongoing diabetes education to be delivered at an individual level with multi-modal education provision.

PAID scores at the initial appointment indicated that 42% might have been in distress about their diabetes care, therefore unable to fully care for themselves. The PAID assessment indicates diabetes related distress [12]. Nicolucci et al. [13] in their study on the psychosocial outcomes of those with diabetes in a global population found 44.6% of participants living with diabetes were in distress about their diabetes care requirements. Due to the distress noted by the people within this present study, it may be beneficial for supporting family to attend appointments. Past studies have also encouraged family participation in education for support and although many family members felt it would be helpful to attend education sessions, less than a quarter actually did so [13, 14].

Few dyads had full agreement at baseline, however agreement amongst the podiatrist and patient increased at the one month follow up post appointment. There was little change noted in full disagreement and there is unknown behavior change relating to its provision. What was recommended was over the two time points. Written educational information has mixed effectiveness at improving diabetes complications knowledge [15]. What was discovered was that education should be simple, relevant, consistent, and repeated to patients with diabetes [16]. The highest correlation between full agreement was between what the patient identified as the key message at the time of the appointment and at one month post appointment. Perhaps this indicates that the patient participant was able to recall what they felt was the key message, whether this was in agreement with the podiatrists or not.

There are a number of limitations of this study. There was a small sample size of patients and podiatrist participants. As the podiatrists volunteered for the study, this potentially introduced response bias of those who were confident in their ability to provide diabetes education. Patient participants did not contribute to their preference of education modalities during the appointments; this may have increased the retention rate if patients chose an education method and provision in a way that was agreeable to their learning style. Education was also verbal, brochures were not used and use of personalised handouts were limited. As only two participants received personalized handouts, it is not possible to infer that receipt of these did or did not make a difference to recall. It is unclear if other forms of education or a combination of education modalities would increase the recall capabilities of the patient group. Finally, as many of the patient participants were regular patients of the health service, they may have received diabetes education from podiatrists and possibly other health providers on a regular basis. This may have resulted in a higher than average baseline knowledge which impacted on retention.

The volume of information provided to people with diabetes and foot complications is unknown and future research should consider exploring the number of messages, how they are delivered in large cohorts of health professionals. This study did not use audio recordings of consultations and coding of subsequent volume data and the level of detail in transcribed appointments may have also provided more information. This style of data collection would be needed however, if investigation of the relationship between the overall information load applied to patients during a consultation and how this influenced whether key messages are retained.

## Conclusion

Diabetes is currently a financial and social burden on society, it is vital that health providers equip their patients with the tools to actively treat their diabetes and prevent diabetes related complications. Education is a vital component of the treatment regime for people with diabetes. Good understanding of diabetes related complications ensures a greater understanding of the disease process, and how to prevent costly, both emotionally and financially, complications of the disease. This study highlights that approximately 50% of patient participants did not hold the same key message following a consultation as their podiatrist.

## Additional file

Additional file 1: Patient Participant Checklist. (DOCX 27 kb)

## Abbreviations

BSL: Blood Sugar Level
MoCA: Montreal Cognitive Assessment
PAID: Problem Areas in Diabetes Questionnaire
SD: Standard Deviation
