# Supplementary material for: People with diabetes foot complications do not recall their foot education: a cohort study
Source: J Foot Ankle Res. 2018 Apr 6;11:12. doi: 10.1186/s13047-018-0255-4 (PMC5889603; doi:10.1186/s13047-018-0255-4)
Supplement: Supplementary file 1 — Patient Participant Checklist. (DOCX 27 kb) [file 13047_2018_255_MOESM1_ESM.docx]

**Additional file 1**

**Patient Participant Checklist**

Identifier No.

DOB:

Male Female

Diabetes Type: 1 2 Years Duration........... Insulin 🞏 OHAs 🞏

Lever of Formal education:

Yr 10 Yr 11 Yr 12 Bachelor Degree Masters PhD

Handouts Provided:

PH diabetes Handout 🞏 Foot care group handout 🞏 Handwritten Information 🞏

Number of podiatry consults in the past 2 years:

1 - 2 3 - 5 6 - 10 More

Content group of oral education given:

Vascular complications 🞏 Neurological complications 🞏

Footwear 🞏 General foot care 🞏

Wound care/first aid 🞏 Follow up care 🞏

Referrals:

Diabetes Education 🞏 Endocrinologist 🞏 Vascular Surgeon 🞏

Dietician 🞏 Other………………………….

Most important information from podiatrist:

…………………………………………………………………………………………………………………………………………………………………………………………………………………………………………………………………………………………………………………………………………………………………………………………………………………………………………………………………………………………………………………………………………………………………………………………………………………………………………………………………………………………………………………………………………………………………………………………………………………………………………………………………………………………………………
